# Expression of Interleukin-8, Interleukin-10 and Epstein-Barr Viral-Load as Prognostic Indicator in Nasopharyngeal Carcinoma

**DOI:** 10.5539/gjhs.v7n3p364

**Published:** 2015-04-23

**Authors:** Eka Savitri, Mubarika Sofia Haryana

**Affiliations:** 1 Department of Ortholaryngology, Faculty of Medicine, Hasanuddin University, Makassar, Indonesia; 2 Department of Biomolecular/Histology, Faculty of Medicine, Gadjah Mada University, Indonesia

**Keywords:** EBV DNA Load, (VCA-p18 + EBNA-1) IgA, IL-8, IL-10, NPC

## Abstract

Interleukin-8 (IL-8) is angiogeneic chemokine that plays a potential role in both development and progression of many human malignancies including nasopharyngeal carcinoma (NPC). Epstein- Barr virus (EBV) is recognized to be an important etiologic agent of NPC as the viral gene products are frequently detected in NPC tissue along with the elevation of antibody titre to the viral protein (VCA-p18+ EBNA1) of IgA in the majority of patients. Elevated plasma of Viral Load is regarded as an important marker for the presence of the disease and for the monitoring of disease progression. However, other serum/plasma parameters such as the level of certain interleukins (IL-8 and IL-10) has also been implicated in NPC progression. The study aimed to investigate the correlations between plasma Viral Load and the level of interleukin (IL-8) and Interleukin (IL-10) in relating these parameters to the stages of NPC. In addition of Viral Load (VCA-p18+EBNA1) IgA, Interleukin-8 and Interleukin-10 before and after therapy will be investigated to seek the possible marker for disease progression. A total of 39 NPC patients and 29 healthy control individuals enrolled in this study. Plasma Viral Load was quantified using real-time quantitative PCR. The Level of plasma interleukins both IL-8 and IL-10 were analyzed using ELISA methods. Results indicated there was a significant decrease in viral load was detected in plasma of NPC patients following therapy. Plasma of viral load was shown to be a good prognosticator for disease progression. There were positive correlation between plasma of viral load and IL-8. These non invasive parameters expressed in blood, could be substitutes of viral load using brushing method, which is invasive. In conclusion that: Viral Load, (VCA-p18+EBNA1) IgA and IL-8 levels are promising markers for the presence of NPC and progression of the disease.

## 1. Introduction

Nasopharyngeal cancer (NPC) is a malignant epithelial neoplasm with incidence, which is commonly attracts the upper tracts of autodigestive. Epidemiology of NPC is very unique, which rarely found in European populations, but many are in China (particularly in southern China), Southeast Asia, and Africa. In Indonesia the incidence rate of NPC is 4.7/100,000 population per year in comparison between men and women of 2-3: 1. Almost 80% of NPC’s patients diagnosed at the age of 30-59 years, Chien & Chen (2003), with a trend of increased incidence with age. Incidence in Makassar of South Sulawesi province from January 2004 to June 2007, obtained 33% of malignancies in the ear, nose and throat (Sung et al., 1998; Punagi & Savitri 2007)

Epstein-Barr virus (EBV) has a closely relationship with the NPC. Viral gene product protein that are detected on the NPC tissue of the majority of sufferers are LMP1 (Latent membrane protein 1), LMP2 (latent membrane protein 2), EBNA 1-6 (Epstein-Barr Nuclear Antigen 1-6), it was also found an increase of immunoglobulin A antibody titers (IgA) viral proteins (viral capsid antigen/VCA and early-antigen/EA) (IgA (VCA-p18 + EBNA-1). Viral Load in Makassar are 866.4 million people while the highest was 48.84 million at Yogyakarta (Eka Savitri, 2007). The differences of highly significant viral load is an interesting question for us and let to encouraged the research considering viral infection would lead to the immune system to be activated. So we assume that the result of EBV infection associated with the occurrence of NPC related to the result of lack of the immune system ability to eliminate the virus as seen from immunologic indicators (Fachiroh et al., 2004; Stevens et al., 2005; Tomokazu et al., 2001).

Virus interactions and secretion of IL-8, found has relation with the activity of Latent Membrane Protein 1 (LMP1). The activity of IL-8 and IL-10 will influence the pathways of angiogenesis and aggressively of disease (Punagi & Savitri, 2007; Fachiroh et al., 2004). LMP1 also shown to stimulate p38 MAPK cascade and the activation of various genes including IL-8 and IL-10. Pathway activation is through activation of CTAR1, 2 (Cytokines Tumor necrosis factor Receptor Activating), TRAF (Tumor necrosis factor Receptor Associated factor), TRADD (TNF Receptor-Associated Death Domain), at the end will affect the JN (Tomokazu et al., 2001; Fachiroh et al., 2005).

K pathways (Jun N kinase) and AP1 (Activating protein) which in turn stimulate IL-8 and IL-10 become more active. Cytokine of IL-8 can be proved to play a role in inflammatory processes, tumor genesis and angiogenesis through micro vessel formation in tumors and metastasis. IL-8 can stimulate endothelial adhesion, trigger transendothelial migration, and activates the neutrophils. In addition, IL-8 can act as a chemo tactic factor to T infiltrating lymphocytes (TIL) so that it will increase its infiltration in NPC tissues (Meichi et al., 2008).

Evidence showed that the role of IL-8 in malignant melanoma was to increase its potential metastatic, but in nasopharyngeal cancer role of IL-8 is still far from clear. Presumably, interaction and internal communication between the downstream (molecular underneath) of LMP1 and CD40 molecules was found to stimulate the expression of JNK and AP1, which then those will stimulate angiogenesis, inflammation, progression and metastasis caused by IL-6 and IL-8 cytokines that produced, Fachiroh, et al., (2004). Some studies indicated that viral load can be used to determine the prognostic of NPC, and it is an important marker for progression as well as a monitor recurrence of NPC patients. Research by (Tan Eng Lai *et al*. 2006) showed that the elevation of EBV DNA load correlated with higher levels of IL-6 and IL10 (Stevens et al., 2005). Because IL-8 and IL-10 are also encouraged through the activation of LMP-1 on the NPC, the viral load, levels of IL-8 and IL-10, can be used in determining the progressivity and metastasis. The combination of all three factors are assumed to have potential markers for prognostic factor of NPC (Tomokazu et al., 2001; Sunil & Govindarajan, 2005; Meichi et al., 2008).

Expression of IL-8, IL-10 and viral load in nasopharyngeal cancer as a prognostic factor has not been studied in Indonesia. If the expression of IL-8 and IL-10 in biopsy tissue correlated with the concentration of IL-8 & IL-10 in the blood, it is assumed that this will be a new innovation as minimal invasive examination that is highly needed to assess the prognostic indicators and progression after therapy. Moreover, if it is known that activation pathway of IL-8 and IL-10 through p38-MAPK can be proven to have role in the pathway of NPC therefore this pathways can be used as biologic targeted of therapy in the treatment of NPC in the future (Sunil & Govindarajan, 2005; Meichi et al., 2008).

## 2. Materials and Methods

The sampling used was purposive method with 39 patients who had been diagnosed with NPC by histopathology examination taken as sample of cases, and 29 healthy people are enrolled in this study as control.

### 2.1 Isolation of DNA by Boom Method

EBV DNA Load in blood is examined with a quantitative light cycler (LC) real time PCR. Primers used are QP1 and QP2, and internal hybridization probes fluorigenic of EBNA LCN and EBNA FLN (TIBMOL Biol, Berlin, Germany), has been described by Stevens. LC-based PCR amplification of 99-bpEBNA1 (location of 213-bp QP1-QP2 amplicon) are used to quantify the small fragments of DNA. In this experiments, reagents and hybridization probes on the 99-bp LC-based PCR-bpLC identical 213-based PCR, except primer (forward primer QP3 15-CCACAATGTCGTCTTACACC-3) and reverse primer QP4 15-ATAACAGACAAFGGACTCCCTL-3J). Real-time PCR reagents from Roche Diagnostics (Almere, The Netherlands). Elisa examination of IgA (VCA - P18 + EBNA 1) by taking blood samples from as 6 ml, 0.5 ml of blood taken + 4.0 ml of lysis buffer N are mixed immediately and stored at -80°C. The remaining blood serum are isolated and stored at -20°C. Serum was analyzed using synthetic peptide epitopes immune dominant proteins and EBNA1 VCA-p18.

ELISA plate coated with a combination of peptides (1 ug/ml of EBNA1 plus 0.5 ug/ml VCA-p18) in 0.05 M Na2CO3, pH 9.6 incubated for two hours at 0°C. After that waste the liquid, give 3% BSA (in 1x PBS) 200 ul/bowl on the layers, then incubate for 1 hour at 37°C, wash three times with PBS Tween 0.05%. Next take 100ul samples (1:100), serum added and incubated for 1 hour at 37°C, cover plate/bowl and discard the liquid. After the fourth washing with PBS-Tween 0.05%, discard the washing liquid. Give conjugate (mous anti-human IgA-HRP dissolved in the liquid sample (1: 4000), plates cover was incubated for 1 hour at 37°C. Discard the liquid, wash with PBS Tween 0.05% (4x), discard the wash liquid. Mix the solution TMB A (red) and B (blue) (1:1), give color to the TMB (100ul/container), incubated in the dark space for 30 minutes. Give 100ul/bowl of 0.5 M H2SO4, avoid waving. Read OD 450 nm by using ELISA reader.

### 2.2 Check of Elisa IgA (VCA – P18 + EBNA 1)

Mix P18 and antigen in plate and incubated at 40°C or 37°C for 2 hours. Supernatant is discarded, add 3% BSA (made in 1x PBS), incubated for 1 hour in 37°C. Supernatant was discarded and then add PBS Tween 0.05% (4 times). Add with mouse anti-human IgA-HRP, incubated for 1 hour 37°C. Supernatant was discarded and then add PBS Tween 0.05% (4 times). Add 100ul TMB to each well, incubated for 30 minutes in dark room. Add 100 ul of 0.5 M H2SO4 into each well and read at a wavelength of 450 nm.

### 2.3 Check Interleukin- 8 (IL-8) by Elisa Using a Quantikine IL-8 Kit

Preparation of samples and reagents. Prepare the plate. Put 100 ul assay diluent RD1-85 into each well. Add 50 ul standards of samples and control into each well and incubated for 2 hours at room temperature. Discard the supernatant and wash with buffer for 4 times. Add 100 ul conjugate into each well and incubated for 1 hour at room temperature. Discard the supernatant and wash with buffer for 4 times. Add 200ul solution substrate into each well and incubated for 30-minutes in dark room. Add 50 ul stop solution into each well and read at a wavelength of 450 nm.

### 2.4 Check of Interleukin -10 (IL-10) by Elisa Using Quantikine HS IL-10 Kit

Preparation of samples and reagents. Put ul assay diluent RD1-10 into each well and add 200 ul standards of samples and control into each well then incubated for 2 hours at room temperature and above the shaker. Discard the supernatant and wash with wash buffer for 6 times. Add 200 ul conjugate into each well and incubated for 2-hours on shaker. Discard the supernatant and then wash with buffer for 6 times. Add 50 ul of substrate solution into each well and incubated for 1 hour at room temperature. Add 50 ul amplifier solution into each well and incubated for 1 hour at room temperature. Add 50 ul stop solution into each well and read at a wavelength of 450 nm.

## 3. Result

The study was conducted to 50 cases of patients with NPC based on AJCC / UICC and with 29 controls not suffering of NPC. Both brushing examination for viral load and viral load is only done to the patient of NPC, the controls are not performed by medical ethical considerations. Blood is checked to assess IgA (VCA-p18 + EBNA1), viral Load, IL-8 and IL-10 in the case of NPC before and after therapy are included in the remaining 39 inclusion criteria and 29 controls.

**Figure 1:**
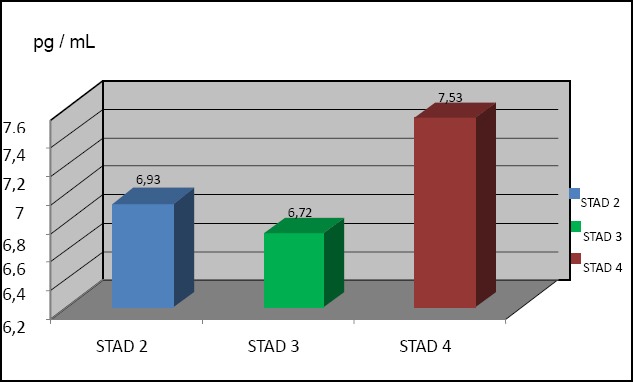
Level of IL-8 based on the stadium of NPC

**Figure 2:**
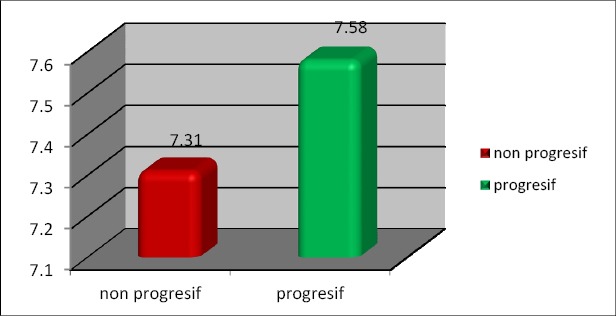
Levels of IL-8 based on progression

Among inflammatory mediators, some cytokines and chemokins such as tumor necrosis factor (TNF), interleukin 1 (IL-1), interleukin 6 (IL-6), interleukin 8 (IL-8) are cytokines that play role in tumor genesis. In the inflammation-like microenvironment (the area around the tumor), the interaction between cytotoxic T cells (CTL) and tumor cells are important in the growth of NPC. The interaction can be mediated by some chemokine atau sitokine. Another way of interaction may involve cell contact through ligand-receptor binding, for example, tumor-infiltrating T cells can provide a survival signal to cells of NPC through CD40-CD40 ligand interaction, prevents tumor cells from CD95-triggered apoptosis. Research by Harijadi *et al*, 2002 has shown a correlation between the high of intra-tum Eka Savitri oral infiltration of T cells (CTL) and poor prognosis of NPC, this study supports the hypothesis that infiltration of T cell can effect the progression of NPC.

Levels of IL-10 in stadium II is relatively higher compared with stage III, but level of IL-10 in stadium III is higher found in stadium IV. In this study we obtained that level of IL-10 is higher found at an earlier stadium then further decreased through late stadium. The results found by Loren Kis *et al*., 2005 shown that in the serum of individuals infected with Epstein-Barr produced IL-10. Epstein-Barr virus also express BCRF1 proteins that have a similar activity with IL-10 referred to as viral IL-10 (v-IL-10). Detection of IL-10 using recombinant IL-10 showed low expression (50 times lowest) and it turns out after investigation it is due to the effect of inhibiting of blocking of antibody and v-IL-10 against IL-10 receptor. It also demonstrated that affinity bond between v-IL-10 and IL-10 R found 1000 times lowest. This may be explained in our study that IL-10 expression in higher stadium given outcome is lower, although at a higher stage, the number of virus copies increase make the production of v-IL-10 will also increase as well. v-IL-10 will then act as a blocking antibody to IL-10 binding to IL-10 receptor.

Bound between the receptor and the v-IL-10, although very weak which has been demonstrated in previous studies (Loren Kis, 2005), but will decrease the levels of v-IL-10 detected by ELISA, otherwise at lower stadium level v-IL-10 found lower (in this case the amount of virus circulating is assumed lower than v-IL-10 than the v-IL-10 then the result checks obtained by ELISA found the higher level of v-IL-10. To ensure the expression of IL-10 in the tissue or circulation it needs to be examined. We assumed that the higher the stage the higher antibody blocking of IL-10.

**Figure 3:**
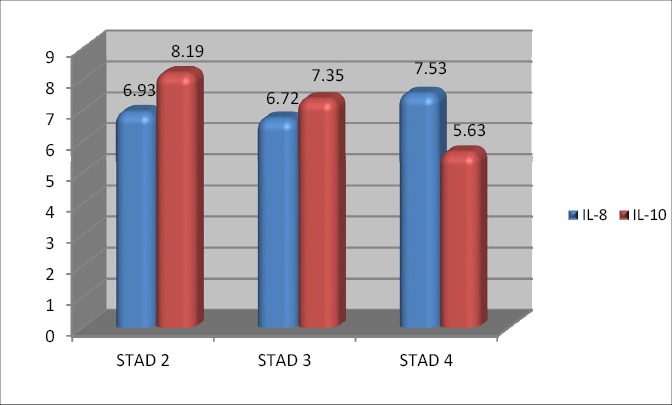
The ratio of IL-8 and IL-10 based on the stadium of NPC

When compared the incidence of progressive we obtained two groups: first group with a ratio of IL-8: IL-10 > 1 and second group with progressive ratio of IL-8: IL-10 <1 is 22.7%: 0%. When the cut of point was used as a cut of points then the ratio > 1 indicates the condition is worse than the ratio <1 in terms of progression. This condition indicates the possibility to use the ratio of IL-8: IL-10 as the ratio which is prognostic that showed progression.

## 4. Discussion

The data taken from Wahiddin Hospital in Makassar South Sulawesi province from January 2004 to June 2007 shown NPC cases reached as 33% of malignancies in the ear, nose and throat. Nasopharyngeal cancer occurs commonly in men, and in the productive age between 35-50 years. However recently found many cases in children and younger people. In addition, research evidence suggests that there are differences related between EBV - Indivividual - the immune system in different geographic area. In the case of DNA viral load research conducted in Yogyakarta showed a different trend with our investigation result. Besides type EBV, polymorphism of EBV genome, HLA Class 1, associated with EBV in Yogyakarta shown the differences of images and results publication elsewhere. In addition, there is still little known of role of cytokines in EBV infection associated with NPC particularly with the prognosis. LMP-1 that is oncogenic shown to play a role in various pathways of internal regulatory of immune system through the NFkB ^(4)^, which in turn related to the stimulation system of humoral immune to produce of antibodies (IgG, IgA against VCA, EA, EBNA’s) (Sunil & Govindarajan 2005) or responses of cellular immune with EBV epitope presentation on MHC class 1 which recognize by CTL. Prognostic marker is needed in addition to VCA-p18 and EBNA-1, our preliminary study proved that the number of copies of EBV DNA viral load is very high in NPC’s cases in Makassar (Savitri, 2006). Based on the specificity of NPC cases in Makassar, we further analyze the relationship between EBV DNA viral load with IgA (VCA-p18 + EBNA -1), IL-8 and IL-10 for use as a prognostic biomarker for NPC in Makassar.

### 4.1 Relationship Between EBV DNA viral Load with Progressifity of NPC

Another factor is why the number of copies in Makassar 40 times fold higher than in Yogyakarta? This is probably caused by the genetic variation in EBV genome so EBV-infected cells can escape from patrolling of immune system such as genetic variation in MHC class 1 epitope at LMP1 and LMP2 (Haryana *et al*., 2000). This research is conducted with 39 NPC’s patients based on histopathology examination and 29 healthy individuals as controls to see the relationship of viral load with disease progression. Examination of viral load was conducted by Real Time PCR (RT- PCR) in the NPC patients using brushing and samples derived from peripheral blood (plasma). Medium of control is only performed using blood samples only.

The results of this study indicates that the blood of DNA viral load (average of NPC cases are 168.220.393; controls are 2673.238 (p = 0.007) demonstrated significant statistically. The results of analysis of the relationship between viral load from NPC’s blood compared to controls showed that viral load of NPC patients are 75 times highest than control. This result showed that the viral load of NPC cases in Makassar has relation with the researchers conducted previously by the team in Yogyakarta, Taiwan, Hong Kong, and Malaysia. To see the possibility of viral load whether can be used to follow up after treatment so *viral load* of NPC cases were also examined before and after therapy. These results showed that the viral load in NPC patients after brushing therapy and blood method showed a significant decrease.

Research in Malaysia by Stevens et al. (2005) also showed a significant reduction of EBV DNA viral load of NPC patients after therapy. Our results concluded that the viral load in plasma may represent brushing both before and after therapy, so check the viral load can be done easier, cheaper and more convenient for patients, especially after therapy. In addition, it was concluded also that EBV DNA of viral load can be used as a good prognostic indicator of disease progression.

### 4.2 Level of IgA (VCA-p18+EBNA-1) on the NPC’s Patient of Makassar

Examination results by ELISA shown that levels of IgA (+ VCAp18 EBNA-1) in 39 cases of NPC and 29 of controls/healthy individuals increased 2000 times higher in cases compared to control. These results are the same with Fachiroh, 2005 in Yogyakarta but IgA (VCA-p18 + EBNA-1) level derived from Makassar samples showed higher average (3.4836 SD 4.4695; *p* = 0.000). While the case in Yogyakarta show average of NPC which are 1.2431 SD 0.9808, so it can be seen that levels of IgA (VCA-p18 + EBNA-1) from the NPC cases of Makassar 4 times higher than in Yogyakarta. Even when it was seen from the increase in the number of copies of DNA viral Load 2000 times higher and IgA is only 4 times higher.

### 4.3 Ratio of IL-8 dan IL-10 in Relation With Progressifity of the Disease

Comparison of progressive events with a ratio of IL-8: IL-10 > 1 compared to non-progressive with a ratio of IL-8: IL-10 are bigger than 1 is 22.7%: 0%. When the cutoff point used as the cut of point then the ratio > 1 indicates the condition is worse than the ratio of <1 in terms of progression. This condition indicates the possibility to use the ratio of IL-8: IL-10 as a prognostic ratio to show the progression (table 4). In Figure 6 can be seen that in patients of NPC stadium 2 and 3 found high levels of IL-10 but at a lower level in stadium 4 can be used as prognostic and follow-up for treatment. If the ratio of IL-8: IL-10 > 1 indicates a bad prognostic, and NPC be progressive, treatment must be re-assessed whether the doses needs to change or replacement the types of drugs given.

**Table 1:** The Characteristic of Sample

Characteristic	Group	
	Cases	Control
**Sex**		
-Men	26 (66.7%)	14 (48.3%)
-Women	13 (33.3%)	15 (51.79%)

**Stadium**		
Stadium 2	5 (12.8%)	
Stadium 3	15 (38.5%)	
Stadium 4	19 (48.7%)	

**Age**	45.32 ± 13.72	27.35 ± 8.60

**Tribe**		
Buginese	16 (41%)	9 (31.03%)
Makassar	10 (25.6%)	8 (27.59%)
Poso	1 (2.56 %)	1 (3.45%)
Mandar	1 (2.56 %)	1 (3.45%)
Toraja	7 (17.95%)	3 (10.34%)
Buton	1 (2.56%)	2 (6.90%)
Tionghoa	1 (2.56%)	2 (6.90%)
Jawa	2 (5.13%)	2 (6.90%)
Bali		1 (3.45%)

**Table 2:** Distribution of cases based on Histopathological status

Status of histopathology (WHO)	n	%
Type I: Carcinoma of squamosa cell with ceratinized	1	26
Type II: Differentiated carcinoma without ceratinized	13	33,33
Type III: Ana plastic	25	64,1

**Table 3:** Analysis of IL-10 levels based on the stage of NPC

Comparing with each stadium	Level of IL-10 (pg/mL)	*p*
Stadium II – III	8,19 (8,01) - 7,35 (5,06)	0.866
Stadium II – IV	8,19 (8.01) - 5,63 (4,47)	0.581
Stadium III – IV	7,35 (5,06) - 5,63 (4,47)	0.179

*Note.* *p* value by U Man Whitney test.

**Table 4:** The ratio of IL-8 and IL-10 based on the disease progression

	Ratio of IL-8/10 > 1	Ratio of IL-8/10≤ 1	Total
**Progressif**	5 (22.7%)	0 (0.0%)	5 (16.13%)
**Non progressif**	17 (77.3%)	9 (100%)	26 (83.87%)
**Total**	22	9	31

*p* = 0,155 (Fisher test)

**Table 5:** Analysis of the differences between *viral load*, IgA (VCA-p18 + EBNA-1), IL-8 and IL- 10 in cases and control

Variables	n	Average	*p*
1. Viral load in blood			
a. Cases	39	168220.393±228488,0035	0.007
b. Control	29	2673.2379 ± 6036,6756	
2. Viral capsid in antigen			
a. Cases	39	2743.7828 ± 4,4695	0.001
b. Control	29	0.2764 ± 0,3396	
3. IL-8			
a. Cases	39	7.141215 ± 0,5471	0.000
b. control	29	2.631917 ± 0,5299	
4. IL-10			
a. Cases	39	6.620713 ± 16676,3289	0.006
b. Control	29	4.048212 ± 6,7049	

## 5. Conclusion

Conclusion of this research are as follow:

1) The ratio of IL-8 and IL-10 can be used to assess the prognosis of NPC. Ratio of IL-8: IL-10 > 1 indicates a poor prognosis.
2) There is a positive correlation between EBV DNA viral load with IL-10.
3) EBV DNA of viral load can be used as a marker for treatment succesfully.
4) EBV DNA of viral load and levels of IL-8 is a marker of NPC and can be used for assessment of treatment result.
5) Plasma of EBV DNA of viral load equivalent with EBV DNA of viral load of from smears Brushing. Therefore, plasma of EBV DNA viral load can be used to follow up with minimal invasive with a relative cheaper cost and as a replacement of brushing of EBV DNA of viral Load.
6) EBV DNA of viral load is a prognostic indicator of disease progression.
7) EBV DNA of viral load in NPC patients from Makassar is very high compared with EBV DNA Viral Load of patients from Yogyakarta. Possible differences in the type of EB virus strains and variations of HLA class 1 and the variation of EBV genome can not be ignore.
8) Further research is still needed to know the differences between the viral load from Makassar and Yogyakarta.

## References

[ref1] Chien Y. C, Chen C. J, Bishop J, Huang P, Johnson P. J, Sham J. S. T, Soo K. C (2003). Epidemiology and etiology of Nasopharyngeal Carcinoma: Gene-Environment Interaction. Cancer Reviews.

[ref2] Decaussin G, Lammali F. S, Tessier M. T (2000). Expression of BARF1 Gene Encode by Epstein Barr-Virus in Nasopharyngeal Carcinoma Biopsies. Cancer Research.

[ref3] Tan Eng-Lai, Selvaratnam G, Kananathan R, Choon-Kook S (2006). Quantification of eipstein-barr virus DNA load, interluekin-6, interleukin-10, transforming growth factor-βI and steam cell factor in plasma of patients with nasopharyngeal carcinoma. BioMed Cancer.

[ref4] Fachiroh J, Bambang H, Harijadi Paramita D. K, Sophia H, Middeldrop J, Yudharto Marlinda A (2005). Non-invasive diagnosis of nasopharyngeal carcinoma: Nasopharyngeal brushings reveal high Epstein-Barr virus DNA load and carcinoma-specific viral BARF1 mRNA. Int. J. Cancer.

[ref5] Fachiroh J, Schouten T, Hariwiyanto B (2004). Molecular Diversity of Epstein –Barr Virus IgG and IgA Antibody responses in nasopharyngeal Carcinoma: A comparison of Indonesian, Chines, and European Subjects. The Journal of Infectious Diseases.

[ref6] Fachiroh J, Schouten T, Hariwiyanto B, Paramita D. K, Harijadi A, Haryanan Sofia M, Middeldorp (2004). Molecular Diversity of Epsein-Barr Virus IgG and Antibody responses in Nasopharingeal Carcinoma: Acomparison of Indonesian, Chinese, and European Subjects. The Journal of Indonesian Disease.

[ref7] Kis L. L, Nishikawa J, Takahara M (2005). The in vitro EBV-infected subline of KMH2, derived from Hodgkin Lymphoma, expresses only EBNA-1: CD40-ligand and IL-4 induce LMP-1 but not EBNA-2. Int J Cancer.

[ref8] Manna Sunil K, Ramesh Govindarajan T (2005). Interleukin-8 Induces nuclear transcription factor-kB through a TRAF6-dependent Pathway. The Journal of Biological Chemistry 2005.

[ref9] Meichi H, Shih-Yi W, Shih-Shin Ch, Ih-Jen S, Ching-Hwa T, Siao-Jing L, Chang Y (2008). Epstein-Barr virus Lytic Transactivator Zta Enhances Activity chemotactic through induction of Interleukin-8 in Nasopharyngeal Carcinoma Cells. The Jour. of Virology.

[ref10] Middledorp J. M, Brink A. A. A. T. P, Brule A. J. C (2003). Pathogenic roles for Epstein Barr Virus (EBV) gene products in EBV-Associated proliferative disorders. Critical Reviews.

[ref11] Punagi A. Q, Eka S (2007). Profile of Nasopharyngeal Carcinoma in Teaching Hospitals' Medical Faculty of Hasanuddin University Period January 2004 - June 2007.

[ref12] Stevens S. J. C, Verkuijlen Hariwiyanto B, Harijadi Fachiroh J, Paramita D. K, Middeldorp Diagnostic Value of Measuring Epstein-Barr (EBV) DNA Load and Carcinoma- Specific Viral mRNA in Relation to anti EBV Immunoglobulin A (Ig A) and IgGAntibody Levels in Blood of Nasophharyngeal Crcinoma Patients from Indonesia. The Journal of Clinical Microbiology.

[ref13] Shao J. Y, Hong L. Y, Liang W. Q (2004). Comparison of plasma Epstein-Barr Virus (EBV) DNA Levels and Serum EBV Immunoglobulin A/Virus Capsid Antigen Antibody Titers in Patients with Nasopharyngeal Carcinoma. American Cancer Society.

[ref14] Sung N. S, Edwards R. H, Moiseiwitch F. S (1998). Epstein Barr Virus Strain Variation in Nasopharyngeal Carcinoma from the endemic and Non-endemic Regions of China. Int. J. Cancer.

[ref15] Sunil K. M, Govindarajan T. R (2005). Interleukin-8 induces nuclear transcription factor-kB through a TRAF6-dependent Pathway. The Jour. of Biol. Chemistry 2005.

[ref16] Tomokazu Y, Toshiyuki H, Ren Q.-ch, Naohiro W, Hajime T, Tzung-Shiahn Sh, Mitsuru F (2001). Induction of Interleukin-8 by Epstein-Barr Virus Latent Membrane Protein-1 and Its Correlation to Angiogenesis in Nasopharyngeal Carcinoma. Clinical Cancer Res.

